# Machine learning-based prediction of early-onset peritoneal dialysis-associated peritonitis: the role of the CONUT score

**DOI:** 10.3389/fnut.2025.1681169

**Published:** 2025-12-11

**Authors:** Hua Zhou, Chunlei Yao, Kai Song, Shuya Zhao, Ye Yuan, Xiangyin Chen, Youqi Ma, Huiyue Hu, Min Yang

**Affiliations:** 1 Department of Nephrology, The Third Affiliated Hospital of Soochow University, Changzhou, Jiangsu, China; 2 Department of Nephrology, The Affiliated Taizhou Second People’s Hospital of Yangzhou University, Taizhou, Jiangsu, China; 3 Department of Nephrology, The Second Affiliated Hospital of Soochow University, Suzhou, Jiangsu, China; 4 Department of Oncology, The Third Affiliated Hospital of Soochow University, Changzhou, Jiangsu, China

**Keywords:** controlling nutritional status, machine learning, peritonitis, peritoneal dialysis, SHapley Additive exPlanations

## Abstract

**Background:**

Peritoneal dialysis-associated peritonitis (PDAP) remains a major complication of peritoneal dialysis (PD). The controlling nutritional status (CONUT) score, which reflects the immune-nutritional state, may offer predictive value in identifying patients at risk. This study aimed to evaluate the utility of machine learning models in predicting early-onset PDAP and to assess the prognostic importance of baseline CONUT score, 6-month CONUT score, and their dynamic changes.

**Methods:**

In this multicenter prospective cohort study, 675 patients initiating PD were enrolled. Multivariable logistic regression was performed to identify clinical predictors of early-onset peritonitis, while Kaplan–Meier survival analysis was used to compare peritonitis-free survival among patients with no peritonitis, early-onset peritonitis, and late-onset peritonitis. To enhance predictive performance, machine learning models including XGBoost, LightGBM, and their ensemble were constructed. Feature selection was based on SHapley Additive exPlanations (SHAP) values derived from an initial XGBoost model. The top 10 SHAP-ranked features were used to train all models. Model performance was assessed using area under the receiver operating characteristic curve (AUC), and SHAP summary plots were generated to interpret feature contributions.

**Results:**

Over a median follow-up period of 41.8 months, 82 patients developed early-onset PDAP. Multivariable logistic regression identified baseline total cholesterol, neutrophil-to-lymphocyte ratio, and 6-month CONUT score as independent predictors of early-onset PDAP (vs. no PDAP; *p* < 0.05). In comparisons between early- and late-onset PDAP, older age, longer PD duration, and lower 6-month CONUT score were independently associated with a decreased likelihood of early-onset PDAP (*p* < 0.05). Using the top 10 SHAP-ranked features, three models (XGBoost, LightGBM, and an ensemble) were trained. For distinguishing early-onset PDAP from no PDAP, LightGBM performed best (AUC = 0.717), followed by the ensemble (0.698) and XGBoost (0.670). In differentiating early- from late-onset PDAP, LightGBM showed the highest AUC (0.781), outperforming the ensemble (0.744) and XGBoost (0.691). SHAP summary plots consistently identified the 6-month CONUT score as the important feature across both classification tasks.

**Conclusion:**

The 6-month CONUT score is an independent predictor of early-onset PDAP and was among the top contributing features in multiple machine learning models. Integrating SHAP-based feature selection with gradient boosting improved model accuracy and interpretability. Dynamic monitoring of nutritional-immune status may aid in early risk stratification and guide personalized prevention strategies in patients undergoing PD.

## Introduction

Peritoneal dialysis (PD) is a common modality of renal replacement therapy, offering patients with end-stage renal disease (ESRD) a home-based, flexible, and cost-effective alternative to hemodialysis (HD). Over the past decades, PD has seen substantial growth globally, particularly in low- and lower-middle income countries, due to its relative affordability and independence from centralized infrastructure (1). Advances in catheter design, dialysate composition, and automated PD systems have further improved its accessibility and clinical outcomes (2). Despite these developments, PD remains constrained by several clinical limitations, the most critical of which is the risk of peritoneal dialysis-associated peritonitis (PDAP). PDAP is one of the most frequent and serious complications of PD, leading to technique failure, hospitalization, catheter loss, and increased morbidity and mortality (3). Early or recurrent episodes can result in peritoneal membrane dysfunction, rendering patients unsuitable for continued PD and necessitating a switch to HD (4).

Traditional risk factors associated with PDAP include poor catheter exit-site care, nasal carriage of *Staphylococcus aureus*, and improper exchange techniques (5). Although these factors guide preventive strategies, they are insufficient for reliably identifying patients at highest risk. Therefore, there is a pressing need for accurate and clinically applicable methods to predict PDAP. Accurate early prediction of PDAP could enable timely interventions, leading to improved clinical outcomes and preservation of peritoneal function. To achieve this, novel biomarkers or risk models capable of stratifying patients based on their susceptibility are required. Compared to traditional statistical methods, machine learning models offer enhanced predictive performance by integrating high-dimensional clinical data, making them valuable tools for individualized PDAP risk stratification. Patients’ nutritional and immune status—and dynamic changes therein—have emerged as important determinants of PDAP risk (6–8). The controlling nutritional status (CONUT) score, a composite index based on serum albumin, total cholesterol (TC), and lymphocyte count, has been validated as a prognostic marker in various clinical settings (9–11). Our previous studies have demonstrated the utility of baseline CONUT score in predicting outcomes in patients undergoing PD (12). However, it remains unclear whether CONUT score measured during follow-up, or temporal changes in CONUT score, offers superior predictive value for PDAP compared to baseline assessments alone.

The present study aimed to compare the predictive value of the baseline CONUT score, the 6-month CONUT score, and changes in CONUT score during the first 6 months of PD in forecasting the development of PDAP. In addition, we applied machine learning models integrated with SHapley Additive exPlanations (SHAP) to enhance risk stratification by identifying the most informative nutritional predictors and interpreting their contributions to early PDAP risk.

## Materials and methods

### Study design and patients

This prospective cohort study included 1,241 patients who initiated PD at the Third and Second Affiliated Hospitals of Soochow University and the Affiliated Taizhou Second People’s Hospital of Yangzhou University between December 1, 2012, and June 30, 2022. Patients were followed until June 30, 2023. Exclusion criteria were as follows: (1) age < 18 years; (2) PD duration < 6 months; (3) presence of active infection; (4) malignant tumors; (5) estimated glomerular filtration rate (eGFR) (CKD-EPI) (13) ≥ 15 mL/min/1.73 m^2^; and (6) missing data on baseline CONUT score, or 6-month CONUT score. A total of 675 patients were ultimately enrolled. Following catheter insertion, all patients or their caregivers underwent standardized PD training and successfully passed competency assessments. Glucose-based dialysate was used in all cases, and none of the patients used automated PD. The study was conducted in compliance with the principles of the Declaration of Helsinki and approved by the Ethics Committee of the Third Affiliated Hospital of Soochow University (approval number: #52/2016). Written informed consent was obtained from all participants in the study.

### Data collection

Baseline demographic and clinical characteristics were collected at the initiation of PD from the electronic medical records of the three participating centers. Collected demographic data included age, gender, duration of PD, body mass index (BMI), and primary kidney disease. Clinical variables included comorbidities such as hypertension, diabetes, cardiovascular disease, smoking status, autoimmunity disease, and glucocorticoids use. Systolic blood pressure (SBP) and diastolic blood pressure (DBP) were also recorded. Laboratory parameters included hemoglobin, platelet count, neutrophil count, lymphocyte count, potassium, sodium, fasting blood glucose (FBG), blood urea nitrogen (BUN), serum creatinine (SCr), uric acid (UA), eGFR, corrected calcium, magnesium, phosphorus, alkaline phosphatase (ALP), bile acid, serum albumin, prealbumin, C-reactive protein (CRP), TC, triglycerides (TG), intact parathyroid hormone (iPTH), and ferritin. Follow-up laboratory indicators included lymphocyte count, serum albumin, and TC. The CONUT score, based on these parameters, was calculated as previously described (11). Baseline CONUT score referred to the value obtained before PD catheter insertion, and the 6-month CONUT score referred to the value assessed within 1 month before or after 6 months of PD initiation. CONUT score change was defined as the 6-month score minus the baseline score. Patients were stratified into two groups based on the median CONUT score: < 4 and ≥ 4. Patients were classified based on the median CONUT score change: those with a change < 0 and those with a change ≥ 0. The systemic immune-inflammation index (SII) was calculated as (platelet count × neutrophil count) / lymphocyte count. The neutrophil-to-lymphocyte ratio (NLR) was calculated as the neutrophil count divided by the lymphocyte count. The causative organism was identified and recorded at the time of PDAP diagnosis. Causative organism categories include Gram-positive, Gram-negative, polymicrobial, fungus, and culture-negative.

### Study outcome

The primary outcome was the first episode of PDAP, regardless of the causative organism. Competing events included transfer to HD, combined PD and HD, kidney transplantation, death, or loss to follow-up. The diagnosis of PDAP was established when at least two of the following three criteria were met: (1) clinical symptoms such as fever, abdominal pain, cloudy dialysate effluent, nausea, or vomiting; (2) peritoneal effluent white blood cell count exceeding 100/mm^3^ with more than 50% neutrophil count; and (3) positive bacterial identification in the peritoneal effluent by culture or Gram staining (14). Early-onset PDAP was defined as the occurrence of PDAP within 12 months of initiating PD, whereas late-onset PDAP was defined as an episode occurring after 12 months.

### Statistical analyses

Statistical analyses were performed using SPSS software (version 26.0, IBM Corp.) and R software. The normality of continuous variables was evaluated using the Kolmogorov–Smirnov test. Only serum albumin was normally distributed and is presented as mean ± standard deviation; all other continuous variables are presented as medians with interquartile ranges, while categorical variables are summarized as frequencies and proportions.

Patients were classified into three groups based on the occurrence and timing of peritonitis: no PDAP, early-onset PDAP, and late-onset PDAP. For group comparisons, one-way analysis of variance (ANOVA) was employed for the comparison of normally distributed variables, whereas the Kruskal–Wallis test was applied to variables that did not follow a normal distribution. The chi-square test was used for categorical variables.

To develop and evaluate predictive models for peritonitis, the dataset was randomly partitioned into training (80%) and validation (20%) sets using stratified sampling to preserve the proportion of outcome classes. Categorical variables were encoded using dummy variables to ensure compatibility with machine learning algorithms, and feature alignment was performed to maintain consistency between the training and validation sets. Scale_pos_weight parameter was applied during model training for class imbalance. Feature selection was performed by identifying the top 10 most predictive features based on SHAP values derived from an initial XGBoost model. These selected features were subsequently used to train XGBoost, LightGBM, and their ensemble model.

Receiver operating characteristic (ROC) curves were generated for all models, with corresponding AUC values displayed on the plots. Model performance was further evaluated using a comprehensive suite of metrics including accuracy, sensitivity, specificity, positive predictive value (PPV), negative predictive value (NPV), F1-score, and Youden index.

Missing data were addressed using variable-type–specific imputation strategies. For continuous variables, missing values were imputed using the predictive mean matching (PMM) method via the multiple imputation by chained equations (MICE) algorithm implemented in the mice package in R. For categorical variables, missing values were imputed using the mode, defined as the most frequently occurring value within each variable.

Kaplan–Meier (K-M) analysis was used to compare survival between subgroups stratified by the 6-month CONUT score (< 4 and ≥ 4) and CONUT score change (< 0 and ≥ 0). Survival curves were generated for comparisons between: (1) no PDAP vs. early-onset PDAP, stratified by 6-month CONUT score; (2) no PDAP vs. early-onset PDAP, stratified by CONUT score change; (3) early- vs. late-onset PDAP, stratified by 6-month CONUT score; and (4) early- vs. late-onset PDAP, stratified by CONUT score change. Group differences were assessed using the log-rank test.

Cumulative incidence function (CIF) analysis was used to account for competing events, and Gray’s test was applied for between-group comparisons. All statistical tests were two-sided, and a *p* value < 0.05 was considered statistically significant.

## Results

### Characteristics of the participants

A total of 675 patients undergoing PD were included in the analysis. Over a median follow-up period of 41.8 months, 253 patients (37.5%) developed PDAP. Patients were categorized into three groups: no PDAP (*n* = 422), early-onset PDAP (*n* = 82), and late-onset PDAP (*n* = 171). Comparisons of baseline demographic and clinical characteristics across groups are summarized in Table 1. Significant differences among the three groups were observed in age, duration of PD, smoking status, baseline SBP, DBP, sodium, CRP, TC, NLR, 6-month albumin, 6-month TC, 6-month CONUT score, and CONUT score change (*p* < 0.05).

**Table 1:** Baseline characteristics of three groups based on the occurrence and timing of PDAP.

Variables	Total (*n* = 675)	No PDAP (*n* = 422)	Early-onset PDAP (*n* = 82)	Late-onset PDAP (*n* = 171)	*p*-value
Age, years	51.0 (40.5, 63.0)	50.0 (38.0, 61.0)	47.5 (39.9, 66.3)	55.4 (44.0, 65.5)	0.001
Gender (%)					0.223
Male	390 (57.8%)	254 (60.2%)	42 (51.2%)	94 (55.0%)	
Female	285 (42.2%)	168 (39.8%)	40 (48.8%)	77 (45.0%)	
Duration of PD, months	41.8 (24.7, 63.0)	36.8 (21.0, 58.1)	38.8 (22.2, 56.4)	55.2 (38.2, 77.1)	<0.001
BMI, kg/m^2^	22.6 (20.5, 24.6)	22.8 (20.6, 24.7)	22.5 (20.0, 23.9)	22.4 (20.3, 24.7)	0.395
Primary kidney disease (%)					0.099
Glomerulonephritis	290 (43.0%)	180 (42.7%)	36 (43.9%)	74 (43.3%)	
Diabetes	79 (11.7%)	48 (11.4%)	6 (11.4%)	25 (14.6%)	
Hypertension	69 (10.2%)	40 (9.5%)	5 (6.1%)	24 (14.0%)	
Other/unknown	237 (35.1%)	154 (36.5%)	35 (42.7%)	48 (28.1%)	
Hypertension (%)	644 (95.4%)	401 (95.0%)	80 (97.6%)	163 (95.3%)	0.603
Diabetes (%)	157 (23.3%)	92 (21.8%)	20 (24.4%)	45 (26.3%)	0.483
Cardiovascular disease (%)	215 (31.9%)	129 (30.6%)	32 (39.0%)	54 (31.6%)	0.322
Smoking (%)	100 (14.8%)	71 (16.8%)	5 (6.1%)	24 (14.0%)	0.041
Autoimmunity disease (%)	25 (3.7%)	13 (3.1%)	6 (7.3%)	6 (3.5%)	0.176
Glucocorticoids use (%)	76 (11.3%)	51 (12.1%)	12 (14.6%)	13 (7.6%)	0.173
SBP, mmHg	153.0 (138.0, 169.0)	153.0 (138.0, 168.0)	161.0 (145.0, 175.3)	151.5 (136.0, 169.0)	0.013
DBP, mmHg	88.0 (79.0, 99.0)	89.0 (79.0, 99.0)	91.0 (80.0, 103.0)	86.0 (79.0, 95.0)	0.036
Hemoglobin, g/dL	8.1 (6.8, 9.5)	8.3 (6.7, 9.7)	7.9 (6.8, 9.2)	8.0 (6.9, 9.2)	0.388
Platelet, ×10^9^/L	160.5 (125.0, 207.0)	160.0 (125.8, 207.0)	160.5 (121.5, 220.0)	161.0 (121.0, 201.3)	0.890
Neutrophil, ×10^9^/L	4.2 (3.3, 5.5)	4.0 (3.2, 5.4)	4.6 (3.6, 5.8)	4.4 (3.2, 5.6)	0.079
Lymphocyte, ×10^9^/L	1.1 (0.8, 1.5)	1.2 (0.8, 1.6)	1.1 (0.8, 1.3)	1.1 (0.8, 1.4)	0.077
Potassium, mmol/L	4.6 (4.1, 5.1)	4.5 (4.0, 5.0)	4.6 (4.1, 5.3)	4.6 (4.2, 5.1)	0.280
Sodium, mmol/L	140.6 (138.0, 142.8)	140.2 (137.4, 142.7)	140.7 (137.7, 142.3)	140.9 (138.8, 143.3)	0.016
FBG, mmol/L	5.1 (4.5, 6.3)	5.0 (4.5, 6.2)	4.9 (4.3, 6.4)	5.2 (4.6, 6.6)	0.253
BUN, mmol/L	29.7 (24.0, 36.9)	29.5 (24.0, 36.5)	31.1 (24.6, 39.1)	29.4 (23.4, 36.9)	0.474
SCr, μmol/L	764.0 (621.0, 960.0)	761.5 (629.0, 951.3)	762.5 (619.0, 968.2)	765.0 (597.0, 981.0)	0.942
UA, mmol/L	482.1 (390.0, 566.7)	478.8 (383.5, 572.5)	472.1 (395.9, 543.3)	489.0 (399.2, 563.0)	0.629
eGFR, ml/min/1.73 m^2^	5.9 (4.6, 7.6)	5.9 (4.7, 7.6)	5.8 (4.5, 7.5)	6.0 (4.2, 7.6)	0.552
Calcium, mmol/L	2.2 (2.0, 2.3)	2.2 (2.0, 2.4)	2.2 (2.1, 2.4)	2.2 (2.0, 2.3)	0.412
Magnesium, mmol/L	0.9 (0.8, 1.1)	0.9 (0.8, 1.1)	0.9 (0.8, 1.1)	1.0 (0.8, 1.1)	0.592
Phosphorus, mmol/L	1.9 (1.6, 2.3)	1.9 (1.6, 2.3)	1.9 (1.5, 2.4)	1.9 (1.6, 2.3)	0.711
ALP, U/L	74.0 (58.0, 94.0)	74.0 (56.5, 95.0)	66.5 (55.5, 88.0)	75.0 (60.0, 96.8)	0.248
Albumin, g/dL	34.4 ± 5.3	34.5 ± 5.1	33.3 ± 4.6	34.9 ± 5.9	0.070
Prealbumin, mg/dL	28.0 (22.4, 32.0)	28.2 (23.0, 32.5)	26.6 (21.6, 32.0)	27.1 (22.1, 31.3)	0.376
CRP, mg/L	5.6 (4.3, 7.4)	5.5 (4.1, 7.3)	5.1 (3.8, 6.3)	6.1 (5.0, 9.8)	<0.001
TC, mmol/L	4.1 (3.4, 4.8)	4.0 (3.4, 4.7)	4.2 (3.5, 5.2)	4.3 (3.5, 5.1)	0.028
TG, mmol/L	1.4 (1.0, 2.0)	1.4 (1.0, 2.0)	1.4 (1.0, 1.9)	1.4 (0.9, 2.0)	0.889
Bile acid, μmol/L	2.7 (1.6, 4.3)	2.6 (1.6, 4.2)	2.3 (1.6, 4.0)	2.9 (1.9, 4.5)	0.126
iPTH, pg/mL	285.0 (170.6, 442.9)	288.5 (171.2, 430.3)	285.0 (168.3, 457.1)	277.4 (170.5, 459.2)	0.995
Ferritin, μg/L	181.0 (84.0, 336.0)	179.6 (82.5, 335.0)	183.4 (81.4, 316.6)	203.3 (94.1, 360.8)	0.878
Baseline CONUT score	4.0 (3.0, 6.0)	4.0 (3.0, 6.0)	4.0 (2.0, 6.0)	4.0 (3.0, 6.0)	0.843
SII	558.5 (376.8, 963.8)	539.8 (365.1, 928.2)	702.9 (424.3, 1097.6)	603.5 (398.0, 1036.0)	0.087
NLR	3.7 (2.5, 5.7)	3.5 (2.4, 5.3)	4.0 (3.0, 6.0)	3.8 (2.6, 6.1)	0.023
Causative organism (%)					0.465
Gram-positive	103 (40.7%)		39 (47.6%)	64 (37.4%)	
Gram-negative	56 (22.1%)		19 (23.2%)	37 (21.6%)	
Polymicrobial	5 (2.0%)		1 (1.2%)	4 (2.3%)	
Fungus	7 (2.8%)		2 (2.4%)	5 (2.9%)	
Culture-negative	82 (32.4%)		21 (25.6%)	61 (35.7%)	
6-month lymphocyte, ×10^9^/L	1.4 (1.0, 1.8)	1.4 (1.1, 1.8)	1.3 (0.9, 1.6)	1.3 (1.1, 1.7)	0.152
6-month albumin, g/dL	34.0 ± 5.9	34.4 ± 5.4	30.9 ± 7.9	34.4 ± 5.7	<0.001
6-month TC, mmol/L	4.3 (3.7, 5.1)	4.3 (3.6, 5.0)	4.3 (3.7, 5.2)	4.4 (3.9, 5.3)	0.037
6-month CONUT score	3.0 (2.0, 5.0)	3.0 (2.0, 5.0)	5.0 (2.8, 7.0)	3.0 (2.0, 5.0)	<0.001
CONUT score change	−1.0 (−2.0, 1.0)	−1.0 (−2.0, 1.0)	0.5 (−2.0, 3.0)	−1.0 (−2.0, 1.0)	0.001

PDAP, peritoneal dialysis-associated peritonitis; OR, odds ratio; CI, confidence interval; PD, peritoneal dialysis; BMI, body mass index; SBP, systolic blood pressure; DBP, diastolic blood pressure; FBG, fasting blood glucose; BUN, blood urea nitrogen; SCr, serum creatinine; UA, uric acid; eGFR, estimated glomerular filtration rate; ALP, alkaline phosphatase; CRP, C-reactive protein; TC, total cholesterol; TG, triglycerides; iPTH, intact parathyroid hormone; CONUT, controlling nutritional status; SII, systemic immune-inflammation index; NLR, neutrophil-to-lymphocyte ratio.

Among patients who developed PDAP, Gram-positive bacteria were the most common causative organisms in the first episode (*n* = 103, 40.7%), followed by Gram-negative bacteria (*n* = 56, 22.1%). By the end of follow-up, 138 patients had transferred to HD, 28 had been treated with combined PD and HD, 54 had undergone kidney transplantation, 117 had died, and 7 were lost to follow-up.

### Univariable and multivariable logistic regression analyses: no PDAP vs. early-onset PDAP

Univariable logistic regression identified multiple factors associated with early-onset PDAP compared to no PDAP, including smoking status, autoimmune disease, baseline SBP, serum albumin, TC, SII, NLR, 6-month lymphocyte, 6-month albumin, 6-month CONUT score, and CONUT score change (*p* < 0.1, Table 2). Smoking was excluded from the final multivariable model because none of the female patients in the cohort reported smoking status, indicating a sex-specific association.

**Table 2:** Univariable and multivariable logistic regression of prognostic factors for early-onset PDAP in patients with no PDAP and early-onset PDAP.

Variables	OR (95% CI)	*p*-value	OR (95% CI)	*p*-value
Age	1.006 (0.990, 1.022)	0.439	—	
Gender (female)	1.440 (0.896, 2.315)	0.132	—	
Duration of PD	0.999 (0.990, 1.008)	0.799	—	
BMI	0.947 (0.877, 1.022)	0.164	—	
Primary kidney disease	0.937 (0.850, 1.031)	0.183	—	
Hypertension	2.095 (0.482, 9.112)	0.324	—	
Diabetes	1.157 (0.665, 2.014)	0.606	—	
Cardiovascular disease	1.454 (0.891, 2.372)	0.134	—	
Smoking	0.321 (0.125, 0.822)	0.018	—	
Autoimmunity disease	2.484 (0.916, 6.737)	0.074	—	
Glucocorticoids use	1.247 (0.633, 2.458)	0.524	—	
SBP	1.011 (1.002, 1.020)	0.022	—	
DBP	1.008 (0.994, 1.022)	0.250	—	
Potassium	1.250 (0.914, 1.710)	0.162	—	
Sodium	0.989 (0.937, 1.044)	0.684	—	
Albumin	0.953 (0.910, 0.999)	0.046	—	
Prealbumin	0.999 (0.996, 1.002)	0.673	—	
CRP	0.993 (0.977, 1.009)	0.365	—	
TC	1.280 (1.041, 1.574)	0.019	1.354 (1.098, 1.670)	0.005
Baseline CONUT score	1.039 (0.930, 1.161)	0.497	—	
SII	1.000 (1.000, 1.001)	0.056	—	
NLR	1.081 (1.015, 1.151)	0.015	1.073 (1.005, 1.146)	0.035
6-month lymphocyte	0.664 (0.421, 1.045)	0.077	—	
6-month albumin	0.907 (0.870, 0.946)	<0.001	—	
6-month TC	1.182 (0.960, 1.455)	0.114	—	
6-month CONUT score	1.226 (1.117, 1.345)	<0.001	1.239 (1.126, 1.364)	<0.001
CONUT score change	1.174 (1.074, 1.283)	<0.001	—	

PDAP, peritoneal dialysis-associated peritonitis; OR, odds ratio; CI, confidence interval; PD, peritoneal dialysis; BMI, body mass index; SBP, systolic blood pressure; DBP, diastolic blood pressure; CRP, C-reactive protein; TC, total cholesterol; CONUT, controlling nutritional status; SII, systemic immune-inflammation index; NLR, neutrophil-to-lymphocyte ratio.

In the final multivariable model, baseline TC [odds ratio (OR) 1.354, 95% confidence interval (CI) 1.098–1.670; *p* = 0.005], NLR (OR 1.073, 95% CI 1.005–1.146; *p* = 0.035), and the 6-month CONUT score (OR 1.239, 95% CI 1.126–1.364; *p* < 0.001) remained independent predictors of early-onset PDAP (Table 2). Sensitivity analyses using the imputed dataset yielded consistent findings, confirming the robustness of the final model.

### Univariable and multivariable logistic regression analyses: early-onset vs. late-onset PDAP

Univariable logistic regression analysis comparing early- and late-onset PDAP identified age, duration of PD, smoking status, glucocorticoids use, baseline SBP, DBP, sodium, albumin, 6-month lymphocyte, 6-month albumin, 6-month CONUT score, and CONUT score change as factors associated with late-onset PDAP (*p* < 0.1, Table 3). These variables except smoking status were included in the multivariable model.

**Table 3:** Univariable and multivariable logistic regression of prognostic factors for late-onset PDAP in patients with early-onset PDAP and late-onset PDAP.

Variables	OR (95% CI)	*p*-value	OR (95% CI)	*p*-value
Age	1.017 (0.998, 1.036)	0.078	1.027 (1.006, 1.048)	0.011
Gender (female)	0.860 (0.508, 1.458)	0.576	—	
Duration of PD	1.027 (1.015, 1.039)	<0.001	1.027 (1.015, 1.040)	<0.001
BMI	1.047 (0.964, 1.138)	0.273	—	
Primary kidney disease	1.058 (0.944, 1.187)	0.333	—	
Hypertension	0.509 (0.106, 2.454)	0.400	—	
DM	1.107 (0.603, 2.034)	0.743	—	
Cardiovascular disease	0.721 (0.417, 1.248)	0.243	—	
Smoking	2.514 (0.923, 6.849)	0.071	—	
Autoimmunity disease	0.461 (0.144, 1.475)	0.192	—	
Glucocorticoids use	0.480 (0.209, 1.105)	0.084	—	
SBP	0.985 (0.974, 0.996)	0.009	—	
DBP	0.976 (0.958, 0.994)	0.010	—	
Potassium	0.919 (0.647, 1.305)	0.636	—	
Sodium	1.087 (1.017, 1.162)	0.014	—	
Albumin	1.057 (1.005, 1.110)	0.030	—	
Prealbumin	1.000 (0.996, 1.003)	0.803	—	
CRP	1.017 (0.995, 1.041)	0.136	—	
TC	0.973 (0.790, 1.200)	0.800	—	
Baseline CONUT score	0.973 (0.866, 1.093)	0.643	—	
SII	1.000 (1.000, 1.000)	0.538	—	
NLR	0.985 (0.926, 1.048)	0.629	—	
6-month lymphocyte	1.532 (0.955, 2.458)	0.077	—	
6-month albumin	1.089 (1.042, 1.138)	<0.001	—	
6-month TC	1.040 (0.828, 1.307)	0.737	—	
6-month CONUT score	0.798 (0.720, 0.885)	<0.001	0.806 (0.721, 0.902)	<0.001
CONUT change	0.814 (0.733, 0.903)	<0.001	—	

PDAP, peritoneal dialysis-associated peritonitis; OR, odds ratio; CI, confidence interval; PD, peritoneal dialysis; BMI, body mass index; SBP, systolic blood pressure; DBP, diastolic blood pressure; CRP, C-reactive protein; TC, total cholesterol; CONUT, controlling nutritional status; SII, systemic immune-inflammation index; NLR, neutrophil-to-lymphocyte ratio.

In the multivariable analysis, older age (OR 1.027, 95% CI 1.006–1.048; *p* = 0.011), longer PD duration (OR 1.027, 95% CI 1.015–1.040; *p* < 0.001), and lower 6-month CONUT score (OR 0.806, 95% CI 0.721–0.902; *p* < 0.001) were independently associated with an increased likelihood of late-onset PDAP (Table 3). Sensitivity analyses using imputed data confirmed these associations.

### Machine learning-based classification of early-onset vs. no peritonitis

**Figure 1:**
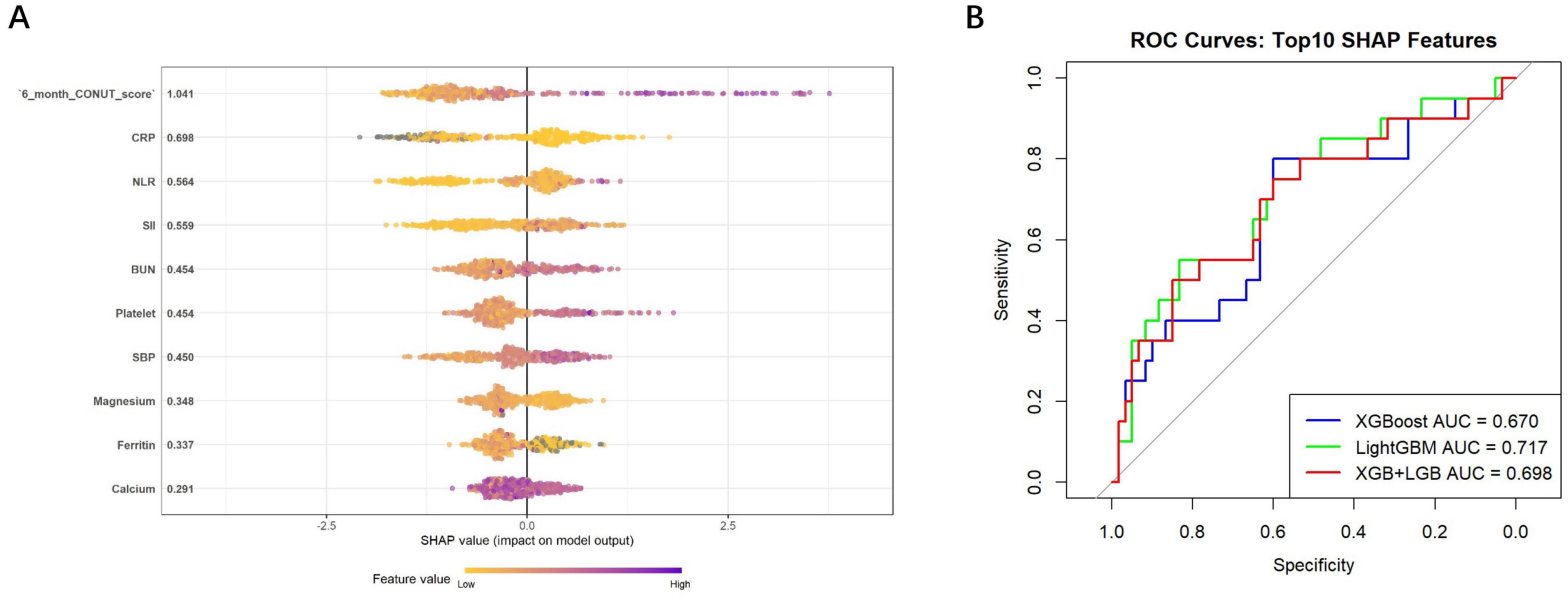
**(A)** Summary plot of SHAP values highlighting the top 10 most influential features for differentiating early-onset from no PDAP; **(B)** ROC curves of three models trained using the top SHAP-ranked features to predict early-onset PDAP. SHAP, SHapley Additive exPlanations; PDAP, peritoneal dialysis-associated peritonitis; ROC, receiver operating characteristic.

To distinguish between patients who did not develop peritonitis and those who experienced early-onset peritonitis, machine learning models were constructed using the top 10 most predictive features identified via SHAP values. As shown in the SHAP summary plot (Figure 1A), the most influential features contributing to model predictions included 6-month CONUT score (SHAP value = 1.041), CRP (0.698), NLR (0.564), SII (0.559), BUN (0.454), platelet count (0.454), SBP (0.450), magnesium (0.348), ferritin (0.337), and calcium (0.291).

Model performance was assessed on the validation set using ROC analysis. Among the three models, LightGBM achieved the highest area under the curve (AUC = 0.717, 95% CI 0.581–0.853), followed by the ensemble model combining XGBoost and LightGBM (AUC = 0.698, 95% CI 0.556–0.841), and the XGBoost model (AUC = 0.670, 95% CI 0.526–0.814) (Figure 1B). Table 4 summarizes the performance metrics of the machine learning models used to differentiate early-onset PDAP from patients without PDAP. These results indicate that LightGBM performing slightly better than the others in distinguishing early-onset peritonitis. However, all three models demonstrated relatively low sensitivity (0.200–0.250), which may limit their ability to identify patients at risk of early-onset PDAP. Conversely, specificity was consistently high across models (0.933–0.950), suggesting good accuracy in excluding non-PDAP cases. Among these models, XGBoost yielded the highest F1-score (0.345) and Youden index (0.183), indicating a more balanced trade-off between sensitivity and specificity.

**Table 4:** Machine learning model metrics for predicting early-onset PDAP vs. no PDAP.

Model	AUC	95%CI	Accuracy	Sensitivity	Specificity	PPV	NPV	F1	Youden index
XGBoost	0.670	0.526–0.814	0.763	0.250	0.933	0.556	0.789	0.345	0.183
LightGBM	0.717	0.581–0.853	0.763	0.200	0.950	0.571	0.781	0.296	0.150
XGB + LGB	0.698	0.556–0.841	0.763	0.200	0.950	0.571	0.781	0.296	0.150

PDAP, peritoneal dialysis-associated peritonitis; AUC, area under the curve; CI, confidence interval; PPV, positive predictive value; NPV, negative predictive value.

### Machine learning-based classification of early-onset vs. late-onset peritonitis

**Figure 2:**
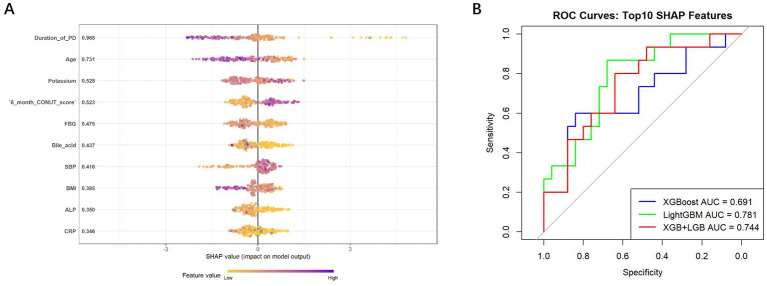
**(A)** Summary plot of SHAP values highlighting the top 10 most influential features for differentiating early-onset from late-onset PDAP; **(B)** ROC curves of three models trained using the top SHAP-ranked features to predict early-onset PDAP. SHAP, SHapley Additive exPlanations; PDAP, peritoneal dialysis-associated peritonitis; ROC, receiver operating characteristic.

To explore the discriminatory features between early- and late-onset peritonitis, SHAP values were used to rank feature importance. As shown in the SHAP summary plot (Figure 2A), the most influential variables in the predictive model included duration of peritoneal dialysis (SHAP value = 0.968), age (0.731), potassium (0.528), 6-month CONUT score (0.523), FBG (0.475), bile acid (0.437), SBP (0.416), BMI (0.385), ALP (0.350), and CRP (0.346).

Three machine learning models were trained using these top 10 features. ROC analysis on the validation set demonstrated that LightGBM achieved the best performance with an AUC of 0.781 (95% CI 0.637–0.925), followed by the ensemble model of XGBoost and LightGBM (AUC = 0.744, 95% CI 0.586–0.902), and XGBoost alone (AUC = 0.691, 95% CI 0.510–0.872) (Figure 2B). The performance metrics of machine learning models for predicting early-onset PDAP vs. late-onset PDAP are presented in Table 5. XGBoost demonstrated higher sensitivity (0.533) and specificity (0.840), together with the best F1-score (0.593) and Youden index (0.373), suggesting a superior balance between sensitivity and specificity.

**Table 5:** Machine learning model metrics for predicting early-onset PDAP vs. late-onset PDAP.

Model	AUC	95%CI	Accuracy	Sensitivity	Specificity	PPV	NPV	F1	Youden index
XGBoost	0.691	0.510–0.872	0.725	0.533	0.840	0.667	0.750	0.593	0.373
LightGBM	0.781	0.637–0.925	0.650	0.467	0.760	0.538	0.704	0.500	0.227
XGB + LGB	0.744	0.586–0.902	0.700	0.533	0.800	0.615	0.741	0.571	0.333

PDAP, peritoneal dialysis-associated peritonitis; AUC, area under the curve; CI, confidence interval; PPV, positive predictive value; NPV, negative predictive value.

### Impact of CONUT score on early-onset PDAP

**Figure 3:**
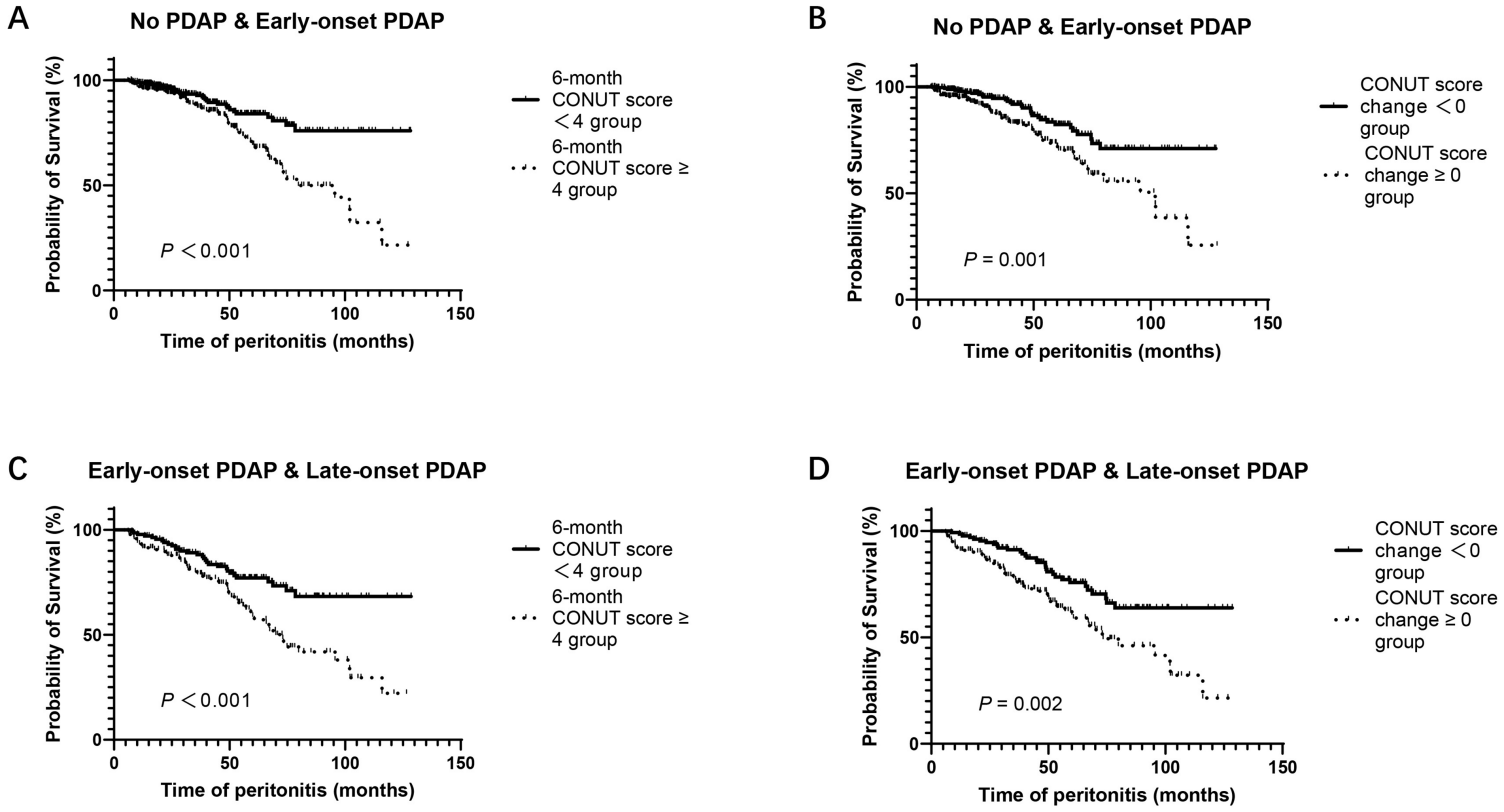
Kaplan–Meier curves for overall survival of PDAP **(A)** No PDAP & early-onset PDAP according to 6-month CONUT score groups **(B)** No PDAP & early-onset PDAP according to CONUT score change groups **(C)** Early-onset & late-onset PDAP according to 6-month CONUT score groups **(D)** Early-onset & late-onset PDAP according to CONUT score change groups. PDAP, peritoneal dialysis-associated peritonitis; CONUT, controlling nutritional status.

Among patients with no PDAP and early-onset PDAP, K-M survival analyses were performed to evaluate the association of the 6-month CONUT score and CONUT score change with the risk of early-onset PDAP. Patients with a 6-month CONUT score < 4 had a significantly lower risk of early-onset PDAP compared to those with a score ≥ 4 (*p* < 0.001, Figure 3A). Likewise, patients with a CONUT score change < 0 exhibited a lower risk of early-onset PDAP than those with a change ≥ 0 (*p* = 0.001, Figure 3B).

In a separate analysis restricted to patients with early- or late-onset PDAP, K-M curves revealed significant differences in the timing of PDAP onset according to both 6-month CONUT score (*p* < 0.001, Figure 3C) and CONUT score change (*p* = 0.002, Figure 3D).

**Figure 4:**
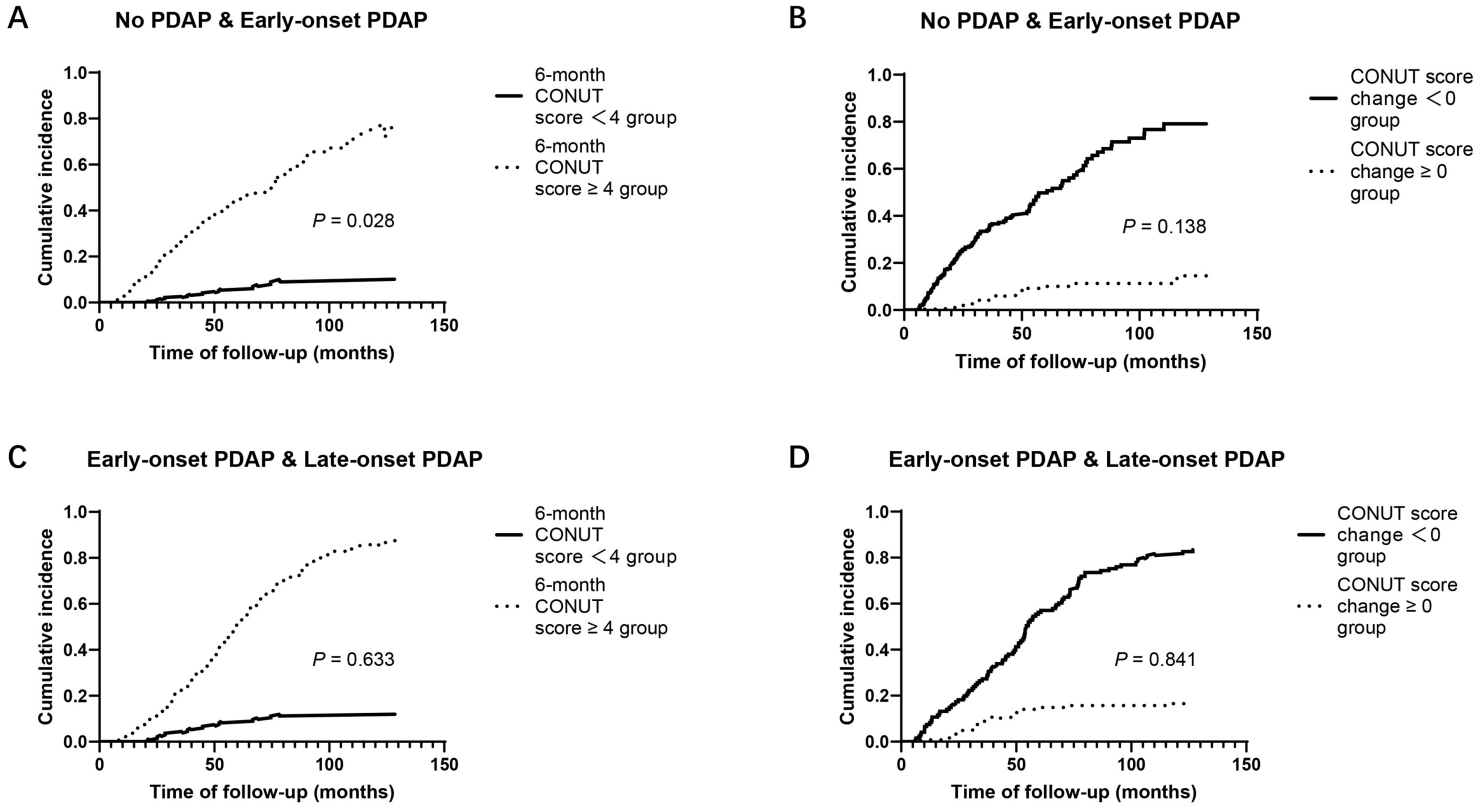
Cumulative incidence function curves of PDAP considering competing risks **(A)** No PDAP & early-onset PDAP according to 6-month CONUT score groups **(B)** No PDAP & early-onset PDAP according to CONUT score change groups **(C)** Early-onset & late-onset PDAP according to 6-month CONUT score groups **(D)** Early-onset & late-onset PDAP according to CONUT score change groups. PDAP, peritoneal dialysis-associated peritonitis; CONUT, controlling nutritional status.

However, in the competing risk analysis using CIF curves among patients without PDAP and those with early-onset PDAP, a significant difference in the cumulative incidence of early-onset PDAP was observed between patients with a 6-month CONUT score < 4 and those with a score ≥ 4 (*p* = 0.028, Figure 4A; *p* = 0.138, Figure 4B). In contrast, no significant differences in cumulative incidence were found when comparing early- and late-onset PDAP according to either the 6-month CONUT score or CONUT score change (*p* > 0.05, Figures 4C,D).

## Discussion

This multicenter study evaluated the prognostic value of the dynamic CONUT score in predicting early-onset PDAP. Multivariable logistic regression identified the 6-month CONUT score as an independent risk factor for early-onset PDAP. K-M analyses revealed significant differences in the incidence of early-onset PDAP among groups stratified by both the 6-month CONUT score and its temporal changes. In addition, machine learning models incorporating the top 10 SHAP-ranked features demonstrated that the 6-month CONUT score was an important predictor for early-onset PDAP, further highlighting its clinical relevance. Among the models, LightGBM achieved the highest predictive performance, supporting the utility of machine learning-based approaches for individualized risk stratification. Taken together, these results underscore the importance of longitudinal nutritional and immunologic assessment, as well as advanced predictive modeling, in identifying patients at elevated risk of PDAP.

In our study, the 6-month CONUT score emerged as an independent predictor of early-onset PDAP. Although each component has previously been associated with outcomes in patients undergoing PD, the composite score offers a synergistic assessment. Serum albumin, a well-established negative acute-phase reactant, serves as a marker for both inflammation and nutritional reserves. Prior studies have demonstrated that low serum albumin correlates with adverse outcomes in PDAP (8). Lymphocyte count reflects immune competence and has been linked to both susceptibility to PDAP and recovery (7). Hypocholesterolemia is frequently observed during systemic inflammatory responses and may reflect the severity of acute illness (15). Previous studies have similarly demonstrated that objective nutritional indexes are associated with technique failure in patients undergoing PD (16). This reinforces the notion that multidimensional scoring systems provide a more comprehensive assessment of patient vulnerability. Our findings support the routine use of the CONUT score as part of risk stratification tools in PD populations and highlight the value of integrated nutritional-immunologic surveillance in clinical practice.

Interestingly, our analysis revealed that the baseline CONUT score—measured prior to the initiation of PD—was not significantly associated with the risk of early-onset PDAP. This finding suggests that pre-dialysis nutritional and immune status may inadequately reflect the dynamic physiological stress and environmental exposures encountered after the initiation of PD. Dialysis initiation marks a profound metabolic and immunologic transition. During the early months of PD, nutritional status and systemic inflammation are highly dynamic and are influenced by factors such as dialysate glucose absorption, peritoneal protein loss, fluid imbalance, and early inflammatory responses (17–20). As such, a static, pre-dialysis assessment may fail to capture the evolving risk landscape following the start of therapy. These findings support the incorporation of serial CONUT assessments—particularly within the first 6 months of PD—as a more meaningful approach to predicting infectious complications.

K-M survival analyses revealed significant differences in early-onset PDAP incidence based on both the 6-month CONUT score and its temporal changes. The 6-month CONUT score—rather than the baseline CONUT score—was recognized as an independent risk factor for early-onset PDAP. Patients with persistently elevated CONUT scores at 6 months were at substantially higher risk, underscoring the clinical relevance of dynamic nutritional assessment over static, baseline evaluation. This finding is consistent with previous studies demonstrating that the increment and trajectory of serum albumin levels, particularly during the early phase of PD, may serve as a more critical determinant of patient outcomes (21). While K-M analyses revealed significant associations between both the 6-month CONUT score and its temporal change with peritonitis risk, only the 6-month CONUT score remained independently predictive in the multivariable logistic regression model. This suggests that changes in the CONUT score over time may reflect overlapping risk information already captured by the absolute score at 6 months. In addition, CIF curves found a significant difference only between patients with 6-month CONUT scores <4 vs. ≥ 4 among those with no PDAP and early-onset PDAP. No significant difference was seen between early- and late-onset PDAP. This discrepancy may result from competing events, which CIF accounts for but K-M treats as censored. These results suggest that the 6-month CONUT score better predicts early-onset PDAP risk, but its ability to distinguish early vs. late-onset cases is limited when considering competing risks.

Furthermore, we applied interpretable machine learning to distinguish peritonitis onset patterns in PD patients, with SHAP analysis identifying key predictors and improving clinical interpretability. SHAP analysis identified the 6-month CONUT score as the most influential predictor distinguishing patients without peritonitis from those with early-onset PDAP. Other top-ranking features—CRP, NLR, and SII—highlighted the role of inflammatory and nutritional disturbances, underscoring the importance of immune-nutritional status in infection risk (22). In distinguishing early- from late-onset peritonitis, dialysis duration and age were the most impactful variables, suggesting that longer exposure and older age are key determinants of infection timing, alongside biochemical markers such as potassium, bile acid, and FBG. SHAP plots enhanced model transparency by quantifying the contribution of individual variables to each prediction. Compared to traditional statistical methods, the machine learning approach offers improved accuracy, automatic feature selection, and better handling of multicollinearity—particularly when combined with SHAP. These advantages highlight its potential for developing clinically applicable tools for early risk stratification and personalized care in PD populations. Moreover, LightGBM demonstrated superior discriminative ability, while XGBoost provided a more balanced performance across sensitivity, specificity, and composite metrics, making it potentially more applicable in clinical practice.

This study has several strengths. Unlike prior investigations that assessed nutritional status at a single time point, our longitudinal analysis revealed that the trajectory of the CONUT score serves as a more robust predictor of early-onset PDAP, underscoring the importance of dynamic nutritional and immunologic monitoring. These findings suggest that patients exhibiting a worsening CONUT trajectory may benefit from early, targeted interventions, such as intensified nutritional support, immunomodulatory therapy, and closer clinical surveillance. The multicenter design enhances the external validity of our findings and supports their applicability across diverse patient populations. In addition, the use of interpretable machine learning models, particularly with SHAP-based explanation, enabled the identification of key predictors and provided insights into variable contributions, enhancing both predictive accuracy and clinical relevance. Moreover, the application of competing risk analysis and sensitivity analyses provide a methodologically rigorous framework for modeling infection risk.

However, this study has several limitations. First, partial missing data for the 6-month CONUT score may have introduced bias in evaluating longitudinal nutritional trends. Second, as an observational study, causal relationships cannot be established, and no interventional strategies were employed to modify nutritional or immune status; therefore, we were unable to assess whether such interventions could reduce the risk of PDAP. Third, important clinical parameters, including peritoneal membrane function and dialysis adequacy, were not included in the analysis and may represent potential confounders. Future prospective studies incorporating these parameters and evaluating the impact of targeted nutritional and immunologic interventions are warranted to confirm and expand upon these findings.

## Conclusion

To our knowledge, this is the first study to demonstrate the prognostic value of both the 6-month CONUT score and its trajectory in predicting early-onset PDAP. Dynamic monitoring of nutritional and immune status may support risk-based prevention strategies to improve outcomes and extend technique survival. Furthermore, machine learning models confirmed the 6-month CONUT score as the strong predictor, with SHAP analysis highlighting the added value of inflammatory and biochemical markers. These findings support the development of individualized prediction tools, and future prospective studies are needed to validate their clinical utility.

## Data Availability

The raw data supporting the conclusions of this article will be made available by the authors, without undue reservation.
